# Effectiveness and cost-effectiveness of a guided internet-based Acceptance and Commitment Therapy intervention (MobACT) for adults with Chronic Pain in Italy: study protocol for a randomized controlled trial

**DOI:** 10.3389/fpsyg.2026.1813251

**Published:** 2026-07-13

**Authors:** Michelle Semonella, Alberto De Luca, Carlotta Rodighiero, Gerhard Andersson, Monica Buhrman, Valeria Donisi, Anna Guerrini Usubini, Giulia Landi, Chiara Manna, Ilenia Pasini, Cinzia Perlini, Giada Pietrabissa, Barbara Segattini, Eliana Tossani, Silvana Grandi, Lidia Del Piccolo, Gianluca Castelnuovo

**Affiliations:** 1 Department of Psychology, Catholic University of Milan, Milan, Italy; 2 Department of Psychology, University of Bologna, Bologna, Italy; 3 Department of Neuroscience, Biomedicine and Movement Science, University of Verona, Verona, Italy; 4 Department of Behavioural Science and Learning, Department of Biomedical and Clinical Sciences, Linköping University, Sweden; 5 Department of Clinical Neuroscience, Karolinska Institute, Sweden; 6 HEI-Lab: Digital Human-Environment Interaction Labs, Lusófona University, Lisboa, Portugal; 7 Department of Health, Education and Technology, Luleå University of Technology, Sweden; 8 Department of Psychology, Uppsala University, Sweden; 9 Clinical Psychology Research Laboratory, IRCCS Istituto Auxologico Italiano, Milan, Italy; 10 Laboratory of Psychosomatics and Clinimatrics (Head Prof. Silvana Grandi), Department of Psychology, University of Bologna, Bologna, Italy; 11 Faculty of Psychology, Vita-Salute San Raffaele University, Milan, Italy

**Keywords:** Acceptance and Commitment Therapy (ACT), Chronic Pain, cost-effectiveness analysis, internet-based intervention, randomized controlled trial

## Abstract

**Background:**

Chronic Pain (CP) affects approximately one in five adults and is associated with emotional distress, disability, reduced productivity and substantial healthcare and societal costs. Acceptance and Commitment Therapy (ACT) has demonstrated effectiveness in improving emotional, behavioral and functional outcomes in individuals with CP, but access to ACT is frequently limited by geographic, organizational and workforce barriers. Internet-based interventions may help overcome these limitations by increasing accessibility, reducing treatment costs and improving scalability.

**Objective:**

This study evaluates the effectiveness and cost-effectiveness of MobACT, a guided internet-based ACT programme for Italian adults with CP. The primary effectiveness endpoint is the post-intervention assessment (T1).

**Methods and analysis:**

This two-arm randomized controlled trial will allocate adults with medically verified CP to either immediate MobACT or waitlist control (1:1). Participants in the waitlist control condition will receive access to MobACT only after completion of the T1 assessment, following the primary between-group comparison. A 6-month follow-up (T2) will be conducted in the intervention group only to examine maintenance of treatment effects. The primary outcome will be pain acceptance, assessed with the Chronic Pain Acceptance Questionnaire. Pain intensity and pain interference will be key clinical secondary outcomes. Additional secondary outcomes include quality of life, sleep quality, central sensitization, pain catastrophizing, psychological flexibility, self-efficacy, coping, anxiety, and depressive symptoms. Weekly assessments of sleep quality and changes in pharmacological treatment will also be collected during the active intervention period. The economic evaluation will compare the two conditions from healthcare and societal perspectives using service utilization data, productivity indicators, and EQ-5D-3L–derived quality-adjusted life years.

**Ethics and dissemination:**

Ethical approval was obtained from the Ethics Committee of Università Cattolica del Sacro Cuore, Milan (Approval No.: 146/24). Results will be disseminated through peer-reviewed publications, international conferences and stakeholder reports for patient organizations.

**Trial registration number:**

ClinicalTrials.gov Identifier: NCT07270588.

## 1 Introduction

Chronic Pain (CP) is pain that persists or recurs for more than 3 months in one or more anatomical regions, and is characterized by significant emotional distress (anxiety, anger/frustration, or depressed mood) and/or functional disability (interference with daily life activities and reduced participation in social roles), which cannot be more accurately explained by other diagnoses (Nicholas et al., 2019). Pain is defined as: “Pain is an unpleasant sensory and emotional experience associated with, or resembling that associated with, actual or potential tissue damage.” (Raja et al., 2020), as reported in the International Classification of Diseases (ICD-11) (Raja et al., 2020). Conditions such as widespread chronic pain, complex regional pain syndrome, primary chronic headache, chronic visceral pain, and chronic musculoskeletal pain affect nearly 20% of the population and represent leading causes of disability worldwide (Landi et al., 2020). CP is associated with reduced quality of life (QoL), decreased work productivity, and increased use of medications and healthcare services, representing a substantial economic and social burden with significant direct and indirect costs (Allegri et al., 2015; Maraschini et al., 2025; Toccaceli et al., 2024; Sirri et al., 2020). Consequently, effective management of CP is crucial. CP is multifactorial: biological, psychological, and social factors all contribute to the pain syndrome (Barke et al., 2022). Therefore, pharmacological and surgical approaches alone are often ineffective in alleviating pain or improving physical and emotional functioning (Landi et al., 2021), highlighting the central importance of psychotherapy for this population (Gasslander et al., 2022). Emotional distress in individuals with CP has been linked to pain-related coping styles (Pasini et al., 2024; Castelnuovo et al., 2018). Feelings of depression, anxiety, anger, and fear are bidirectionally associated with the patient's pain experience (Cattivelli et al., 2018; Castelnuovo et al., 2016). According to the fear-avoidance model, these factors influence pain intensity and pain-related disability, together with processes such as pain catastrophizing (i.e., a cluster of maladaptive cognitions and emotions arising in response to pain states) and pain-related fear, through avoidance behaviors (Giusti et al., 2017). Avoidance behaviors, such as activity restriction, may lead to physical deconditioning and further reduced QoL, producing a self-perpetuating vicious cycle (O'Carroll et al., 2026; Hill, 2019; Turk and Rudy, 1992). In this context, treatments based on Acceptance and Commitment Therapy (ACT) aim to reduce the disabling influence of pain by promoting psychological flexibility (PF), defined as the ability to remain open and responsive to present-moment experiences—including pain and discomfort—while engaging in behaviors guided by personally meaningful values (Hayes et al., 2012). This flexible approach involves a willingness to experience pain-related distress in the service of valued life pursuits, fostering engagement in meaningful activities and improving overall pain management (Schütze et al., 2018). Through increased PF, individuals can modify rigid behavioral patterns of avoidance or control that often exacerbate suffering, thereby adapting more effectively to the fluctuating challenges associated with chronic pain (Kashdan and Rottenberg, 2010). Within ACT, PF is operationalized through six interrelated processes—acceptance, cognitive defusion, present-moment awareness, self-as-context, values clarification, and committed action—that together enhance adaptive functioning, resilience, and QoL in the face of persistent pain (Landi et al., 2021; Hayes et al., 2012; Pakenham et al., 2020). ACT can be delivered in brief and modular formats, making it widely applicable, feasible, well tolerated, and effective. As a transdiagnostic approach, ACT targets core processes underlying human suffering, making it potentially suitable for all CP conditions (Feliu-Soler et al., 2018; Scott et al., 2016). Evidence shows that ACT-based treatments effectively reduce pain intensity, pain interference, and emotional distress, while improving patients' QoL (Hughes et al., 2017; Veehof et al., 2016). However, access to these programs is often limited by healthcare system constraints, shortages of trained professionals, lack of service proximity, stigma associated with psychological treatment, and patients' mobility difficulties (Heapy et al., 2015; Perlini et al., 2020). To improve the accessibility and cost-effectiveness of psychological interventions, recent years have seen the development of delivery modes complementing face-to-face treatment. Among these, internet-based self-help interventions grounded in the principles of cognitive-behavioral therapy (CBT) have proven promising for the treatment of chronic pain and related disorders (Ariza-Mateos et al., 2021; Buhrman et al., 2016; Gandy et al., 2022; Macea et al., 2010; Donisi et al., 2023; Pasini et al., 2023). Internet delivery may help overcome many access barriers (De Lucia et al., 2024). Advantages of internet-based interventions (IBIs) include reducing therapist time per patient, shortening waiting lists, enabling patients to work at their own pace, eliminating the need to schedule appointments with a therapist, increasing accessibility for a greater number of people, and potentially improving cost-effectiveness (Andersson et al., 2009; Cuijpers et al., 2008; Semonella et al., 2022). IBIs for chronic pain management has been shown to reduce pain, depression, anxiety, and disability, while improving QoL (Eccleston et al., 2014). More recently, several studies have implemented IBIs based on ACT principles (Bendelin et al., 2020; Buhrman et al., 2013; Lin et al., 2017; Trompetter et al., 2015). Evidence demonstrates that Internet-based ACT Interventions can impact pain acceptance, anxiety, depression, catastrophizing, pain interference, affective distress, pain intensity, pain-related disability, and fear-avoidance, with effect sizes ranging from small to large (Vugts et al., 2018). Despite the growing body of evidence supporting the effectiveness of ACT-based internet interventions for CP patients, no internet-based programme has ever been developed or formally evaluated for the Italian population.

### 1.1 Objectives

The present study aims to examine the effectiveness and cost-effectiveness of MobACT, a guided internet-based Acceptance and Commitment Therapy (ACT) intervention adapted for the Italian adult Chronic Pain (CP) population from a Swedish programme previously tested in adults with chronic pain (Buhrman et al., 2013).

Consistent with the ACT theoretical framework and with the originally registered outcome hierarchy, the primary effectiveness outcome is pain acceptance at the post-intervention assessment (T1). Pain intensity, pain interference, quality of life, sleep quality, central sensitization, pain catastrophizing, psychological flexibility, self-efficacy, coping strategies, anxiety, and depressive symptoms are included as secondary outcomes, as they represent clinically meaningful indicators of symptom burden, emotional adjustment, and daily functioning.

In addition, the study will investigate potential moderators and mediators of treatment effects to better understand for whom MobACT may be most effective and through which ACT-related processes therapeutic change may occur.

The specific study objectives are:

1. To examine the effectiveness of MobACT compared with a waitlist control group at the post-intervention assessment (T1) in terms of pain acceptance;
2. To examine between-group differences at T1 in secondary clinical and psychological outcomes, including pain intensity, pain interference, quality of life, sleep quality, central sensitization, pain catastrophizing, psychological flexibility, self-efficacy, coping strategies, anxiety, and depressive symptoms;
3. To investigate baseline and process-related variables as potential moderators and mediators of treatment effects;
4. To evaluate the cost-effectiveness of MobACT compared with the waitlist condition at T1.

The 6-month follow-up assessment (T2) will be used to evaluate the maintenance of treatment effects within the intervention group. No between-group effectiveness or cost-effectiveness comparisons will be conducted at T2, as participants allocated to the waitlist condition will receive access to the intervention following completion of the post-intervention assessment (T1).

## 2 Methods

### 2.1 Study design

This study is a multicenter two-arm, parallel-group randomized controlled trial (RCT) coordinated by the Department of Psychology, Università Cattolica del Sacro Cuore, Milan, Italy, with collaborating sites at the Università degli Studi di Verona, Verona, Italy, and Alma Mater Studiorum—Università di Bologna, Bologna, Italy, and conducted in collaboration with Linköping University, Linköping, Sweden, and Uppsala University, Uppsala, Sweden.

**Figure 1:**
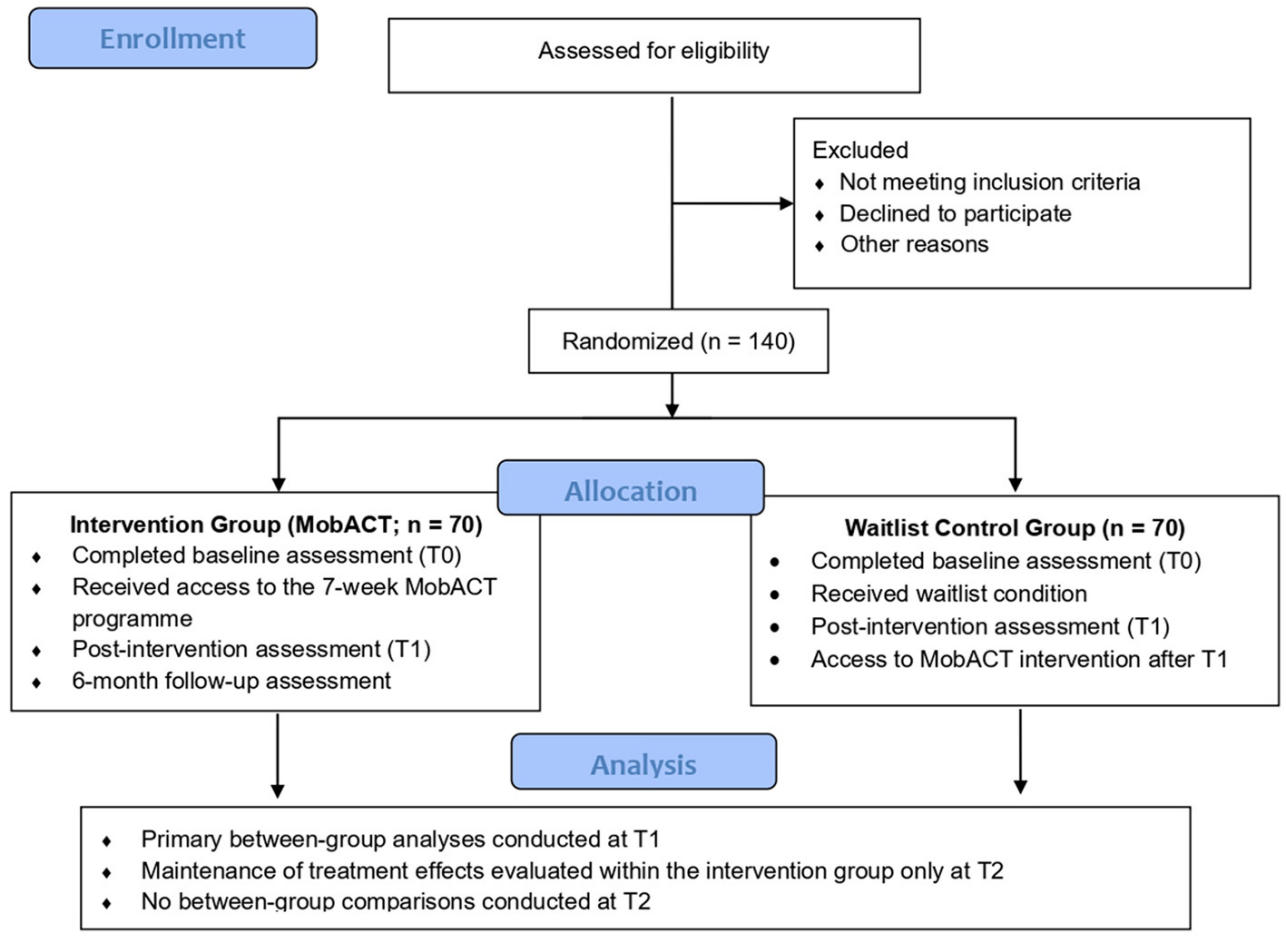
CONSORT flow diagram. As this manuscript describes a study protocol, participant flow is presented prospectively. Reasons for discontinuation, withdrawal or loss to follow-up will be collected at the time the event is identified. through a brief platform message, email or phone contact from a member of the research team. Reasons will be recorded using predefined categories (e.g., time constraints, technical difficulties, symptom worsening, perceived lack of usefulness, preference not to continue, adverse event, or other reason) and will be reported descriptively in the participant flow diagram whenever provided.

Participants will be randomized in a 1:1 ratio to either immediate access to MobACT or to a waitlist control condition. Participants allocated to the waitlist control condition will complete the baseline (T0) and post-intervention (T1) assessments without access to MobACT and will receive access to the programme only after completion of T1, following the primary between-group comparison. Because of this delayed-intervention design, valid between-group comparisons are restricted to T1. The 6-month follow-up (T2) will be conducted in the intervention group only to evaluate maintenance of treatment effects. Informed consent will be obtained from all participants before any study-related procedures are conducted (Figure 1 presents the study flow diagram).

This study will be conducted in compliance with the Declaration of Helsinki and with all relevant national regulations governing research involving human participants. Ethical approval has been obtained from the Ethics Committee of Università Cattolica del Sacro Cuore, Milan (Approval Number: 146/24).

### 2.2 Participants

Individuals will be recruited using digital and community-based dissemination strategies, including:

1. Collaboration with patient associations related to chronic pain (e.g., national and regional organizations), which will share study information through newsletters, websites, social media channels, and member meetings;
2. Word-of-mouth activity using the snowballing sample technique, supported by clinicians, researchers, and community networks who will inform individuals potentially eligible for the study;
3. Online outreach, including the circulation of digital flyers and posts through social media platforms and thematic online groups focusing on chronic pain and psychological wellbeing.

Recruitment materials will provide standardized information regarding the study objectives, intervention characteristics, eligibility criteria, and participation procedures.

Subjects will be eligible for inclusion if they provide informed consent electronically and meet the following criteria: (a) age ≥ 18 years; (b) verified medical diagnosis of chronic pain persisting for at least 3 months; (c) access to an internet-connected device; (d) adequate digital literacy and ability to use online platforms independently; and (e) fluency in Italian.

Exclusion criteria will include: (a) current participation in psychological or psychotherapeutic treatment specifically targeting chronic pain; (b) elevated suicide risk, defined as a score ≥ 1 on item 9 of the Patient Health Questionnaire-9 (PHQ-9; “Thoughts that you would be better off dead or of hurting yourself in some way”) and/or a score ≥ 1 on item 15 of the Web Screening Questionnaire (WSQ; “Have you recently thought about harming yourself or taking your own life?”); (c) self-reported cognitive or neurological impairments potentially interfering with study participation; and (d) diagnosis of severe psychiatric conditions, including psychotic disorders, bipolar disorder with unstable manic or hypomanic episodes, severe depressive disorders, or severe personality disorders significantly affecting daily functioning.

Individuals reporting suicidal ideation will be contacted by a trained therapist, provided with information about available mental health services, and advised seeking help from their general practitioner, the local Mental Health Center or emergency services.

### 2.3 Sample size calculation

The required sample size was determined using G^*^Power software (version 3.1.9.7) for an F test (ANOVA: repeated measures, within-between interaction), with the following parameters: effect size f = 0.15, alpha = 0.05, power (1-beta) = 0.95, two groups, repeated measurements, correlation among repeated measures = 0.50 and no sphericity correction for the primary two-time-point contrast. The assumed effect size was deliberately conservative. Although psychological and online ACT interventions for chronic pain often produce small-to-moderate or moderate effects on pain acceptance and pain interference (Buhrman et al., 2013; Lin et al., 2017; Trompetter et al., 2015; Vugts et al., 2018; van de Graaf et al., 2021; Vlaescu et al., 2016), a smaller effect was selected to avoid overestimating treatment effects in the first formal evaluation of an Italian internet-based ACT programme for CP and in a heterogeneous, fully online recruited sample. The analysis indicated a required total sample size of *N* = 116.

To account for potential attrition, the planned sample size has been increased to 140 participants. This inflation allows approximately 17% attrition while retaining an analyzable sample close to *N* = 116. The assumption is broadly consistent with attrition observed in the Swedish guided internet-delivered ACT trial on which MobACT is based, in which 15 of 76 randomized participants (19.7%) did not provide post-treatment data (Buhrman et al., 2013). Automated reminders, weekly notifications and the availability of therapist contact through the platform are expected to support retention in the present trial.

### 2.4 Procedure

**Figure 2:**
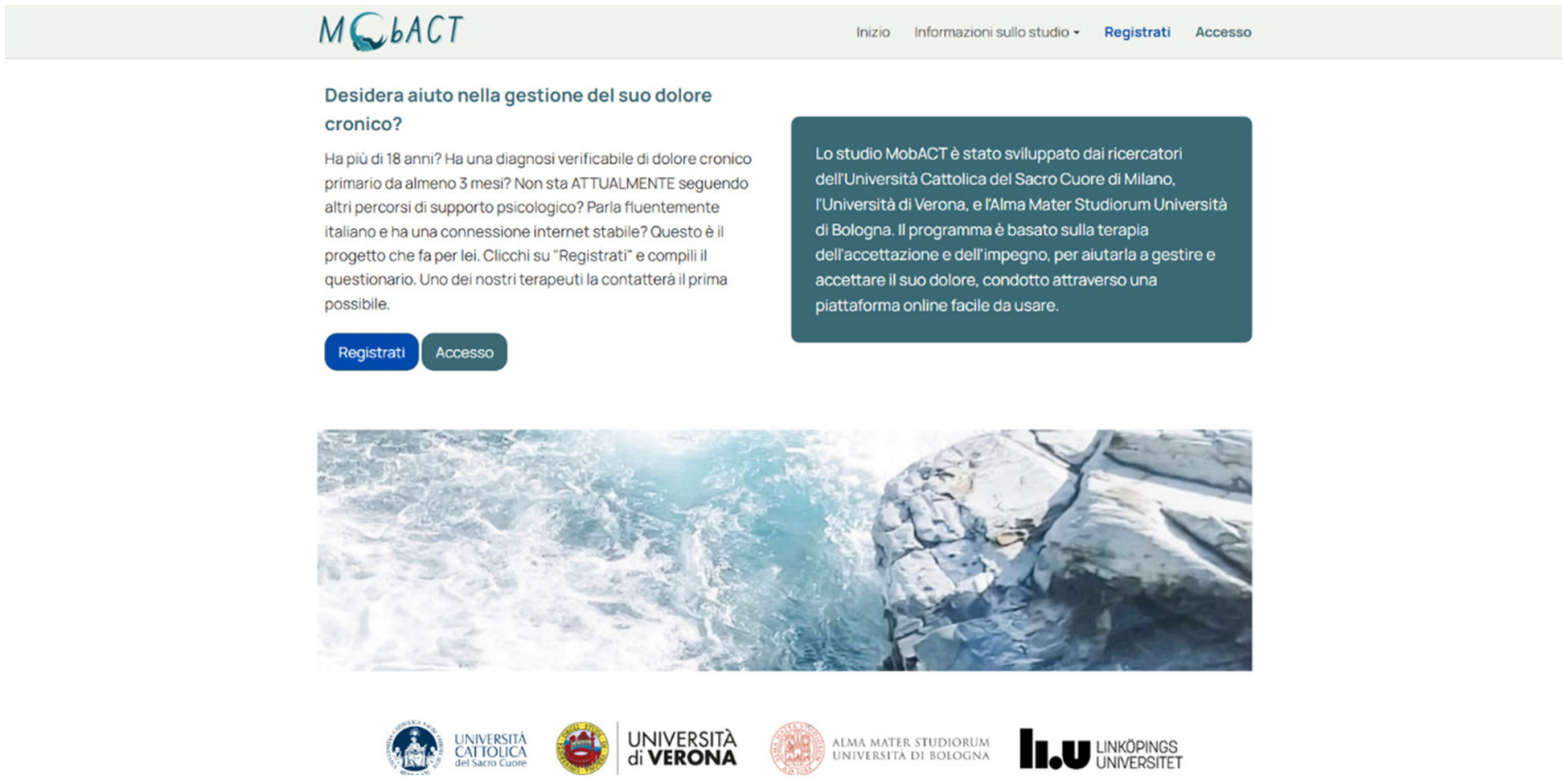
MobACT homepage. The homepage provides an overview of the project, including the research team, study aims, eligibility criteria, and a brief description of the MobACT intervention. It also includes access to the registration section for participant enrolment. Reproduced with permission from MobACT.

Individuals interested in participating will be guided via a secure hyperlink to the MobACT study platform (https://www.iterapi.se/sites/mobact/login) (Figure 2).

After providing informed consent, potential participants will complete an online screening assessment using standardized questionnaires (e.g., Web Screening questionnaire, WSQ) to determine preliminary eligibility. Moreover, within 5 days, they will attend a 30-min semi-structured clinical interview conducted by a licensed clinical psychologist not involved in the study, further assessing eligibility and clarifying study procedures. Eligibility decisions will be then jointly reviewed by the clinician and three principal researchers (Authors M.S., A.D.L. and C.R.). Individuals will be informed of the decision via email within a week.

In cases of exclusion, a brief explanation of the decision will be provided. Individuals presenting severe psychological distress will be encouraged to seek appropriate professional support.

Randomization will occur after eligibility confirmation and completion of the baseline assessment. The allocation sequence will be computer-generated and implemented within the *Iterapi* platform, thereby ensuring allocation concealment until assignment. Due to the nature of the intervention, participants and clinicians involved in screening and intervention delivery cannot be blinded to group allocation.

Eligible participants will then be randomly allocated to either the intervention group, receiving the MobACT programme immediately or the control group (waitlist), receiving access to the programme after 7 weeks.

Participants will receive an email containing their username and a personalized link to create a password. Personal information will be anonymized by assigning each participant a unique identification code. Access to the intervention will be provided through a secure personalized login area on the *Iterapi* platform.

The *Iterapi* platform, developed at Linköping University, is a reliable system designed to deliver internet-based psychological interventions and to support research activities (Semonella et al., 2024a,b). The platform's architecture emphasizes security, process optimization and flexibility, and it supports multiple treatment modalities, including text-based communication, audio and video interactions (Vlaescu et al., 2016).

The platform is designed to be intuitive and user-friendly, ensuring seamless experience across devices such as desktop computers, tablets and smartphones.

*Iterapi* ensures data security and confidentiality through encrypted data transmission and secure storage. Study data is isolated, and access is restricted to authorized personnel only.

The *Iterapi* platform will be the main channel for communication between mental health professionals and participants, as well as for delivering the intervention and collecting quantitative assessments.

**Figure 3:**
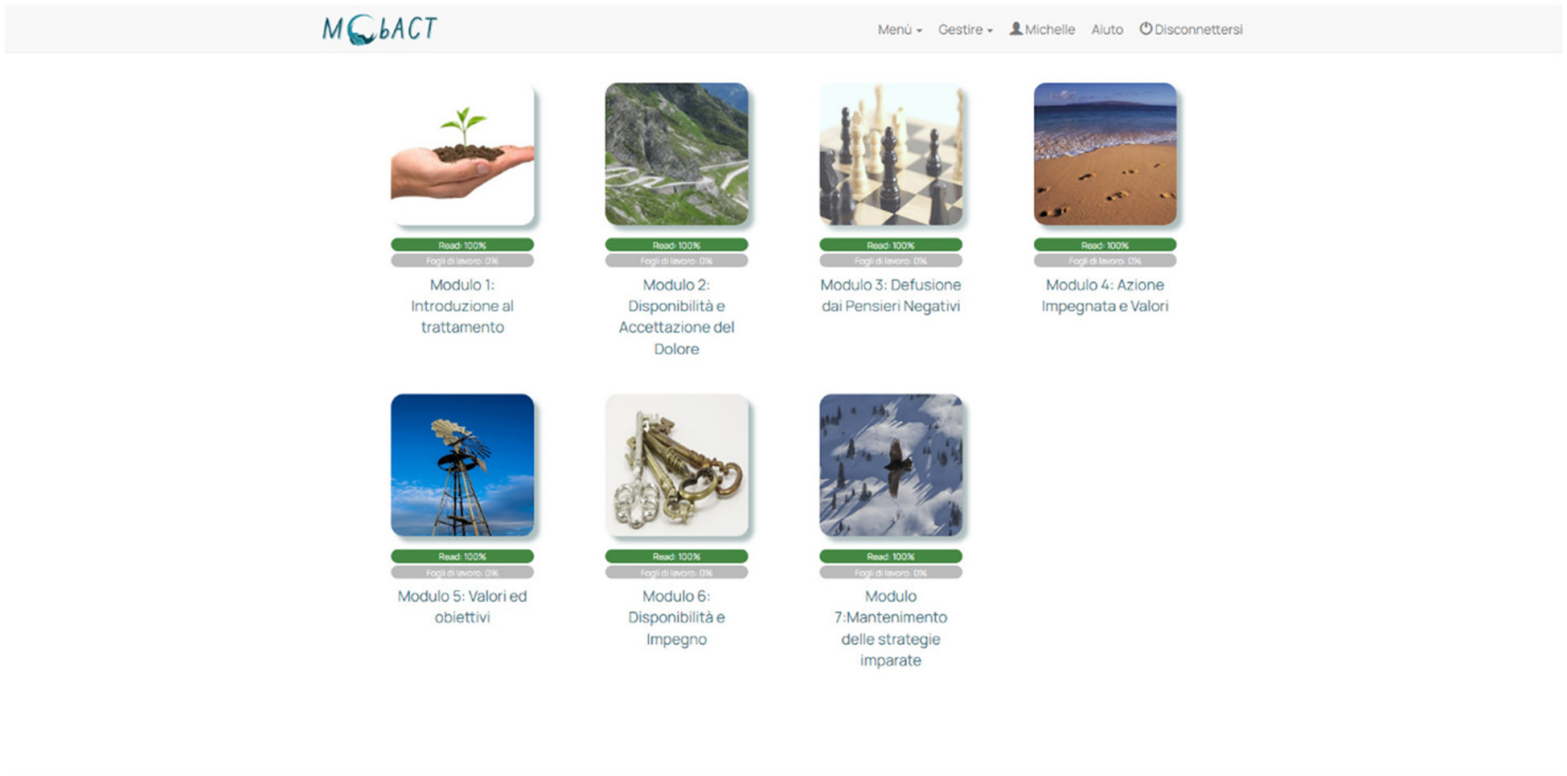
Overview of the MobACT intervention modules. This figure illustrates the seven-module structure of the MobACT programme, outlining the sequence and thematic content of each weekly component of the intervention. Reproduced with permission from MobACT.

**Figure 4:**
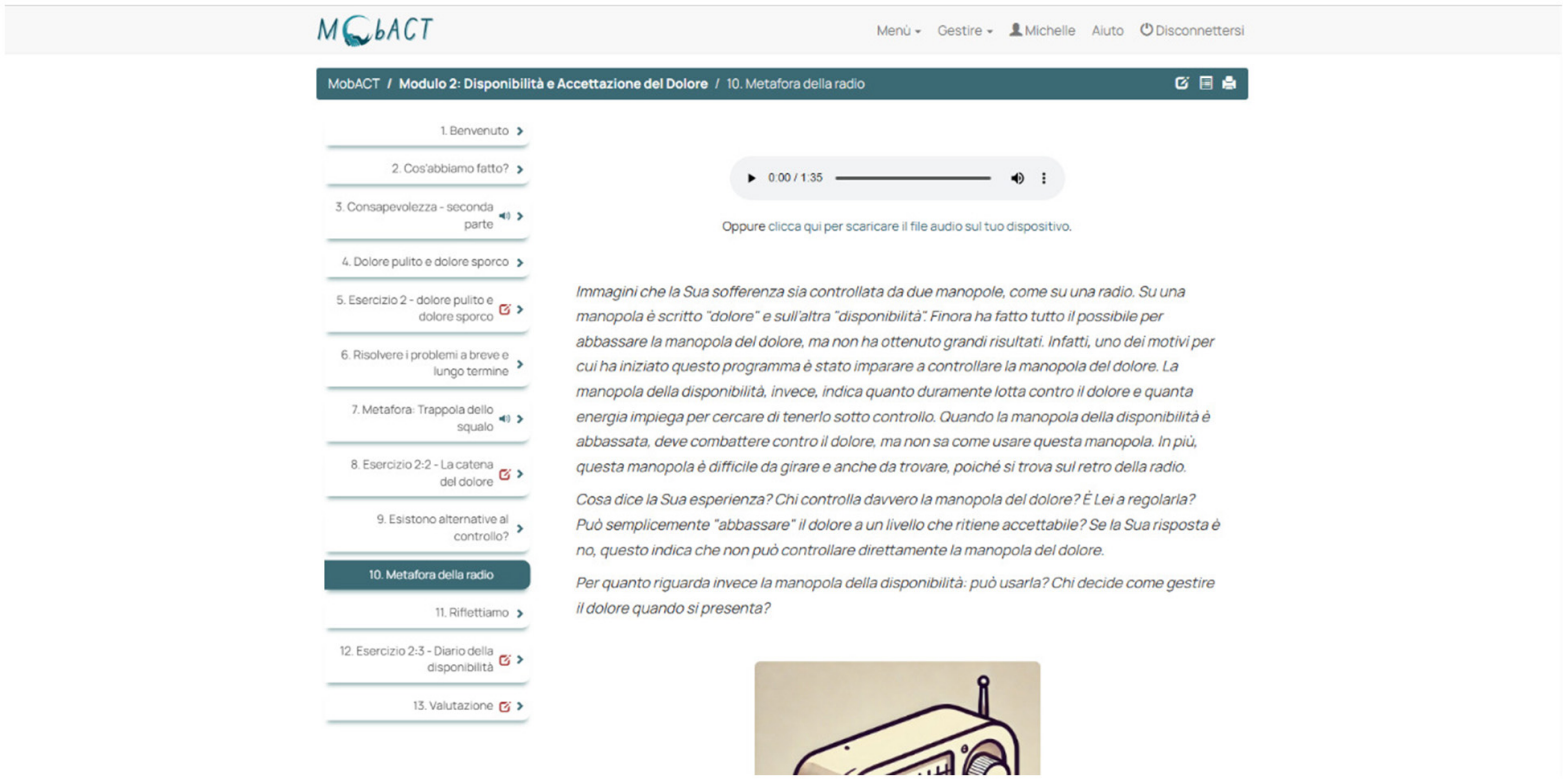
Module 2 – Chapter 10. Example of an ACT metaphor provided in the modules in both textual and audio form. This figure presents one of the ACT metaphors included in Module 2, Chapter 10 of the MobACT programme. The metaphor is available to participants in both written and audio formats, allowing them to engage with the material in the modality that best suits their preferences and learning style. This dual-format delivery aims to support accessibility and facilitate comprehension of key ACT concepts, particularly those related to willingness and acceptance. Reproduced with permission from MobACT.

The treatment consists of seven modules (Figure 3), each addressing a specific component of psychological intervention based on ACT principles. The programme follows the procedures recommended by Hayes, Luoma (Hayes et al., 2006) and has been adapted from a Swedish ACT-based internet treatment that has already been tested and validated in previous clinical studies (Buhrman et al., 2013). Specifically, the content and functionalities of the program were tailored to the Italian general adult population and subsequently assessed for relevance, accessibility, and usability. The module topics are as follows:

1. **Introduction to the treatment:** This module introduces the concept of creative hopelessness. Participants are encouraged to reflect on their past attempts to control pain and to recognize the ineffectiveness of these efforts. This creates a foundation for acceptance and commitment to new strategies. The “Man in the Hole” metaphor is used to illustrate these ideas.
2. **Willingness and Acceptance of Pain:** Participants learn to distinguish between primary and secondary pain. The concepts of willingness and acceptance are introduced. Metaphors such as the “Shark Trap” and the “Radio Metaphor” are used to illustrate the difference between short-term control and long-term acceptance (Figure 4).
3. **Defusion from Negative Thoughts:** This module focuses on mindfulness and cognitive diffusion techniques aimed at helping participants distance themselves from negative thoughts and emotions. The “Bus Metaphor” is used, in which thoughts and emotions are conceptualized as passengers on a bus driven by the participant.
4. **Committed Action and Values:** Participants explore their personal values and learn to cultivate a meaningful life despite the presence of pain. Exercises such as the “Chessboard Metaphor,” which distinguishes between the self and inner psychological content, as well as additional mindfulness practices, are used to facilitate value clarification.
5. **Values and Goal Setting:** Building on the previous module, this section helps participants define and engage in actions aligned with their values. Exercises such as the “Values Compass” and metaphors including “The Skier” and “My Party” support this process.
6. **Willingness Exercises:** This module provides a range of exercises designed to enhance participants' willingness to experience pain without attempting to control it. The “My Party” metaphor is used to demonstrate mindfulness in daily life and committed action.
7. **Maintenance of Learned Strategies:** The final module includes the creation of a maintenance plan and a review of the goals established throughout the programme. It summarizes the entire intervention and offers guidance on how to sustain the strategies learned. Mindfulness practices are emphasized as a central component of the long-term maintenance plan.

Throughout the programme, participants will receive a weekly email every Thursday notifying them that a new module is available on the platform. Participants who do not access the material or complete the assigned exercises will receive a reminder email on Monday, including a brief standardized supportive message aimed at promoting clarification, encouragement, and engagement with the ACT-based activities.

At the end of the programme, participants in both groups will complete the post-intervention outcome assessments (T1), including measures of pain acceptance, pain intensity, pain interference, PF, psychological symptoms, sleep quality, coping strategies, self-efficacy, central sensitization, QoL, and anxiety and depressive symptoms. During the intervention period, participants will also complete brief weekly assessments within the platform, including a visual analog scale (VAS) evaluating perceived sleep quality and a brief item assessing changes in pharmacological treatment for chronic pain. If treatment changes are reported, participants will be asked to specify the nature of these modifications. The platform will allow real-time monitoring of missing or incomplete data, enabling the research team to send reminders or request clarification when necessary.

Following completion of T1 assessments, participants allocated to the waitlist control condition will receive access to the intervention, whereas participants in the experimental group will additionally complete follow-up assessments at 6 months post-treatment (T2).

No adverse or unintended effects related to trial participation are anticipated. However, participants experiencing psychological discomfort will be able to contact the research team for support. Any report of such difficulties will be reviewed by a trained clinician within the research team, who will evaluate whether follow-up is required. In the case of significant clinical concerns, particularly those related to suicidal ideation or risk, the established safety protocol will be applied, involving direct contact with the participant and referral to proper healthcare services. Participants may also contact the psychologist at any time in case of difficulties with the intervention material, questions regarding the exercises, or requests for further clarification. No scheduled therapist sessions will be provided during the programme. Participation is voluntary, and participants may withdraw from the study at any stage without consequences for their future care or treatment.

Throughout the trial, regular internal meetings will be held every 3 weeks to review recruitment progress, adherence levels, technical aspects of data acquisition and any issues arising during implementation. Deviations from the protocol, if any, will be documented, evaluated and reported according to ethical requirements and best practices for clinical research transparency. Upon completion of the trial, all monitoring activities and the integrity of the dataset will be reviewed before proceeding with final analysis.

### 2.5 Qualitative component

Following completion of the intervention and the T1 assessment, approximately 20 participants from the intervention group will be invited to take part in a brief semi-structured interview exploring their subjective experience of the treatment programme and the digital platform (see Supplementary file 1). The qualitative component is included to complement quantitative findings by providing an in-depth understanding of participants' experiences, perceived usefulness of the intervention, barriers and facilitators to engagement, and overall treatment acceptability. Participants will be purposively sampled to maximize variation in relevant characteristics when possible, including age, sex, pain duration and level of intervention engagement. Interviews will be audio-recorded with consent and transcribed verbatim.

### 2.6 Measures

During the initial screening phase, participants will complete the WSQ online (Pietrabissa et al., 2024). The WSQ consists of 15 items assessing a broad range of mental health problems, including depression, generalized anxiety disorder (GAD), panic disorder, agoraphobia, specific phobias, social phobia, post-traumatic stress disorder (PTSD), obsessive–compulsive disorder (OCD), alcohol misuse and suicidal ideation.

Developed at Vrije Universiteit Amsterdam, the WSQ is a brief internet-based tool designed to rapidly identify individuals at risk for common mental disorders. It requires approximately 2 min to complete and is used in this study to ensure adherence to inclusion and exclusion criteria. The WSQ effectively discriminates between individuals with and without psychiatric conditions; however, a positive screen requires further diagnostic evaluation (Pietrabissa et al., 2024). The Italian validation demonstrated good internal consistency (Cronbach's α = 0.732–0.891). Test–retest reliability showed high specificity for several disorders, including agoraphobia (0.947), anxiety (0.959) and panic disorder (0.973), as well as moderate sensitivity values.

At baseline, participants will provide self-reported demographic and clinical information, including age, gender, educational level, and marital status.

In addition, the Italian versions of the following psychological measures will be administered at baseline (T0), post-intervention (T1), and at the 6-month follow-up assessment (T2; intervention group only):

#### 2.6.1 Primary outcome

**Chronic pain acceptance**. Chronic Pain Acceptance will be assessed using the Chronic Pain Acceptance Questionnaire (CPAQ) (McCracken et al., 1999) measuring the extent to which individuals are willing to experience pain while engaging in meaningful activities. The 20-item are rated on a 7-point scale ranging from 0 (“Never true”) to 6 (“Always true”). The questionnaire comprises two subscales: Activity Engagement (AE), reflecting participation in daily activities despite pain, and Pain Willingness (PW), assessing the degree to which individuals refrain from attempts to control or avoid pain. Subscale scores are summed to derive a total score ranging from 0 to 120, with higher scores indicating greater levels of pain acceptance. The Italian validation of the CPAQ reported good internal consistency for the Activity Engagement subscale and total score (Cronbach's α > 0.80), and acceptable reliability for the Pain Willingness domain (α > 0.70) (Bernini et al., 2010).

#### 2.6.2 Secondary outcomes

**Pain intensity and pain interference**. The Brief Pain Inventory (BPI) (Cleeland, 1989) consists of 11-item divided into two subscales. The first subscale assesses pain severity (Severity score, BPI-SS) through four Numerical Rating Scales ranging from 0 (“no pain”) to 10 (“worst imaginable pain”), evaluating current pain, worst pain, least pain and average pain over the past 24 h. The second subscale measures pain interference (Interference score, BPI-IS) with daily activities, walking ability, mood, sleep, work, relationships with others and enjoyment of life, using Numerical Rating Scales ranging from 0 (“does not interfere”) to 10 (“completely interferes”). The BPI-SS is calculated as the mean of four severity items, and the BPI-IS as the mean of seven interference items. Therefore, the maximum possible score for both dimensions is 10. The Italian version of the Brief Pain Inventory has demonstrated good internal consistency, with Cronbach's alpha coefficients ranging from 0.77 to 0.91 across severity and interference subscales, as well as good test–retest reliability, supporting its use in Italian chronic pain populations (Caraceni et al., 1996).

**Quality of life**. The EuroQol-5D-3L (EQ-5D-3L) (Group, 1990) is a standardized instrument developed to measure QoL. The questionnaire consists of two parts. The first part includes five dimensions: mobility, self-care, usual activities, pain/discomfort and anxiety/depression. Each dimension is divided into three severity levels ranging from 1 (“no problems”) to 3 (“extreme problems”). Health states are converted into utility index scores using country-specific value sets, with higher scores indicating better perceived health. The second part includes a VAS indicating current perceived health status on a scale from 0 (“worst imaginable health”) to 100 (“best imaginable health”). The Italian version demonstrated good reliability, with Cronbach's alpha = 0.73 (Savoia et al., 2006).

**Sleep quality**. The Mini Sleep Questionnaire (MSQ) (Zomer, 1985) assesses sleep quality, considering both sleep and wakefulness factors. The questionnaire consists of 10-item, which are rated on a Numerical Rating Scale from 1 (“Never”) to 7 (“Always”) to indicate how well it describes the participant's experience during the previous week. Higher scores indicating poorer sleep quality. Subscale scores were computed for sleep and wakefulness domains, with established clinical cut-offs of >16 and >14, respectively. The Italian version of the MSQ showed good internal consistency (Cronbach's alpha = 0.77), good test–retest reliability (ICC = 0.82) and a factor analysis confirming two dimensions (sleep and wakefulness), both with Cronbach's alpha = 0.75. Optimal cut-off values (>16 for sleep and >14 for wakefulness) have been identified, with an area under the curve greater than 0.80 (Natale et al., 2014).

**Central sensitization**. The Central Sensitization Inventory (CSI) (Mayer et al., 2012) measures current health symptoms related to central sensitization. The instrument includes 25-item rated on a 0 (“never”) to 4 (“always”) scale, yielding a total score ranging from 0 to 100, with higher scores indicating greater symptom severity. A cut-off score of ≥40 has demonstrated good sensitivity and specificity in identifying individuals with central sensitization syndromes. The CSI also includes a supplementary checklist (Part B) assessing prior diagnoses of central sensitization–related conditions (e.g., fibromyalgia, irritable bowel syndrome, migraine). These items are not included in the total score but provide descriptive clinical information. The Italian version demonstrated good internal consistency (Cronbach's alpha = 0.87), ranging from 0.86 to 0.87 if an item is removed (Chiarotto et al., 2018).

**Pain catastrophizing**. The Pain Catastrophizing Scale (PCS) (Sullivan et al., 1995) is used to assess the level of catastrophic thinking associated with pain. The scale consists of 13-item rated on a 5-point Likert scale, where 0 indicates “not at all” and 4 indicates “always”. The PCS includes three subscales reflecting rumination, magnification, and helplessness. The total score ranges from 0 to 52, with higher scores indicating greater levels of pain catastrophizing. A commonly adopted clinical cut-off is ≥30, indicating clinically relevant levels of catastrophizing. The Italian version of the PCS has demonstrated excellent internal consistency (Cronbach's alpha = 0.92) (Monticone et al., 2012).

**Psychological flexibility**. The Multidimensional Psychological Flexibility Inventory (MPFI-24) (Grégoire et al., 2020; Landi et al., 2026) is a self-report instrument consisting of 24-item measuring the six core processes of PF (12-item) and the six processes of psychological inflexibility (12-item). Items are rated on a 6-point Likert scale ranging from 1 (“Never true”) to 6 (“Always true”), referring to experiences over the previous 2 weeks. Flexibility processes include acceptance, cognitive defusion, present-moment awareness, self-as-context, values, and committed action, whereas inflexibility processes include experiential avoidance, cognitive fusion, lack of present-moment awareness, self-as-content, lack of values clarity, and inaction. Global scores for psychological flexibility and inflexibility can be derived. The Italian version of the MPFI has shown excellent internal consistency (Cronbach's alpha = 0.94 for both global psychological flexibility and inflexibility), with subprocesses ranging from 0.85 to 0.94 (Landi et al., 2021, 2026).

**Self-efficacy**. The Pain Self-Efficacy Questionnaire (PSEQ) (Nicholas, 2007) will be used to assess individuals' confidence in performing activities despite pain. It consists of 10-item are rated on a 7-point Likert scale from 0 (“Not at all confident”) to 6 (“Completely confident”), producing total scores ranging from 0 to 60, with higher scores reflecting greater perceived self-efficacy. The Italian version of the PSEQ demonstrated excellent internal consistency (Cronbach's alpha = 0.94) and good test–retest reliability (ICC = 0.82) (Chiarotto et al., 2015).

**Coping**. The Chronic Pain Coping Inventory (CPCI) (Romano et al., 2003) evaluates the frequency of cognitive and behavioral coping strategies used during the previous week via 42-item. The questionnaire comprises eight subscales reflecting distinct coping domains, including guarding, resting, asking for assistance, relaxation, task persistence, exercise and stretching, coping self-statements, and seeking social support. Each subscale yields scores ranging from 0 to 7, with higher scores indicating more frequent use of the respective coping strategy. The Italian version has shown good internal consistency (Cronbach's alpha = 0.71–0.80) and reliability (ICC = 0.70–0.85 and >0.85 across all subscales) (Monticone et al., 2013).

**Anxiety**. The Generalized Anxiety Disorder scale (GAD-7) (Spitzer et al., 2006; Löwe et al., 2008) assesses symptoms of anxiety and counts on 7-item. Each item describes a specific symptom experienced over the past 14 days and is rated on a four-point scale: 0 (“Not at all”), 1 (“Several days”), 2 (“More than half the days”) and 3 (“Nearly every day”). Total scores range from 0 to 21 and reflect anxiety severity. Established severity cut-offs are 5 (mild), 10 (moderate), and 15 (severe anxiety), with scores ≥10 indicating clinically significant anxiety. The Italian GAD-7 has demonstrated good internal consistency (Cronbach's alpha = 0.918) (Ivziku et al., 2019).

**Depression**. The Patient Health Questionnaire (PHQ-9) (Kroenke and Spitzer, 2002) is a self-report instrument made up of 9-item used to assess depressive symptom severity. It screens eight psychological disorders, distinguishing between threshold DSM-IV diagnoses (major depressive disorder, panic disorder, other anxiety disorder, bulimia nervosa) and subthreshold conditions characterized by fewer symptoms than required for formal diagnosis (other depressive disorder, probable alcohol abuse/dependence, somatoform disorder, and binge eating disorder). Participants indicate how often each symptom has occurred over the past 2 weeks on a scale from 0 (“Not at all”) to 3 (“Nearly every day”), resulting in a total score from 0 to 27. Severity thresholds are 5 (mild), 10 (moderate), 15 (moderately severe), and 20 (severe depression), with scores ≥10 typically indicating clinically relevant depressive symptomatology. Item 9 assesses passive thoughts of death or self-harm and will be monitored according to trial safety procedures. The Italian version of the PHQ-9 has demonstrated good internal consistency (with Cronbach's alpha coefficients ranging from 0.86 to 0.92) (Mazzotti et al., 2003).

At each of the seven intervention weeks, participants will complete a VAS assessing their perceived sleep quality for that week (“How would you rate the quality of your sleep during this week?”), ranging from 0 (“worst imaginable sleep quality”) to 100 (“best imaginable sleep quality”).

Participants will also answer a weekly question regarding potential changes in their pharmacological treatment for chronic pain management.

- **Week 1:** “Compared with what you reported at the pre-intervention assessment, have there been any changes in your pharmacological treatment for chronic pain management?”
- **Subsequent weeks:** “Compared with the previous week, have there been any changes in your pharmacological treatment for chronic pain management?”

Response options will be: “No, there have been no changes” or “Yes, there have been changes.” If changes are reported, participants will be prompted to specify the type of modification: “increased dosage of the same medication(s),” “reduced dosage of the same medication(s)” or “administration of different medication(s).”

### 2.7 Cost-effectiveness analysis

To conduct the cost-effectiveness analysis, data will be collected from the beginning of the programme to the final follow-up, and the resources required to deliver the intervention itself will also be calculated. Service use by patients will be recorded through the MobACT platform, covering the use of additional treatments in hospitals and centers, other prescribed therapies, hospital admissions, emergency department visits, outpatient appointments, service utilization, medications and assistive devices.

To enable economic analysis, the questionnaires conducted through the *Iterapi* platform will also collect information on days of work missed, recreational activities, private expenses (e.g., privately paid therapies, travel costs for treatment), informal care, welfare benefits and lost work hours (including those of family members and friends) required to manage the pain condition. The cost-effectiveness analysis will be conducted from both a healthcare perspective and a societal perspective, following existing guidelines. The Second Panel on Cost-Effectiveness in Health and Medicine recommends that all studies report a reference case analysis based on the healthcare sector perspective and another reference case analysis based on the societal perspective.

The Impact Inventory (Sanders et al., 2016), which lists the health and non-health effects of an intervention, makes the economic evaluation more explicit and transparent. For the healthcare sector perspective, the total programme cost per participant will be calculated using the resource-costing method, which includes personnel, supplies and equipment costs (e.g., web hosting service, software maintenance) needed to deliver MobACT. For the societal perspective, the cost of patient time, transportation costs and the cost of unpaid productivity loss due to illness will be calculated.

Specifically, to estimate resource use and costs, targeted questions were formulated by adapting and implementing the questionnaire designed for adolescents with Chronic Pain—parent report (Client Service Receipt Inventory for Adolescents with Chronic Pain–Parent Report) (Sleed et al., 2005), which was used as a reference. This inventory is an adaptation of the CSRI (Beecham and Knapp, 2001) specifically for parents of adolescents with Primary Chronic Pain. The Client Service Receipt Inventory (CSRI) (Beecham and Knapp, 2001) is a tool for estimating direct and indirect costs and is essential in health and health service economics research. It systematically collects data on service use and associated costs, providing a detailed account of the various services utilized by individuals.

The effectiveness of the intervention will be measured based on changes in Quality-Adjusted Life Years (QALYs) and patient productivity (work hours and income). Data collected from the EQ-5D-3L can be converted into a single summary index value, which is essential for calculating QALYs. QALYs are a measure that combines both the quality and quantity of life lived. This metric is used to assess the value of medical interventions by considering the number of life years added by the intervention, adjusted for the quality of those years (Gallego et al., 2024). Each health state described by the EQ-5D-3L has an associated utility value, typically ranging from 0 (equivalent to death) to 1 (perfect health). By multiplying the time spent in a given health state by its utility value, QALYs provide a standardized method for comparing the effectiveness of different treatments and informing healthcare decision-making. One year in a state of “perfect health” equals 1 QALY, whereas being deceased equals 0 QALYs. The formula for calculating QALYs is as follows: number of years of treatment multiplied by the quality-of-life coefficient. For each dimension of the EQ-5D-3L, a score is assigned and then multiplied together to obtain the quality-of-life coefficient, which will be multiplied by the number of years. These data will be collected at pre-intervention (T0), post-intervention (T1) and 6-month follow-up (T2; intervention group only). The use of the EQ-5D-3L to derive QALYs ensures a robust and consistent approach to evaluating and comparing health outcomes across patient populations and different treatments.

### 2.8 Data analysis

All quantitative analyses will be conducted using recognized statistical software packages, including SPSS, Jamovi or R. Prior to carrying out the main analyses, the dataset will undergo systematic cleaning procedures to ensure accuracy and completeness. This process will include the identification of missing or inconsistent responses, the examination of outliers and the verification of the logical coherence of participants' data across time points. When suitable, missing data will be handled using established statistical approaches suitable for longitudinal trials, such as multiple imputation or mixed-effects modeling, in accordance with current methodological recommendations. The primary endpoint for between-group comparisons is the post-intervention assessment (T1). The 6-month follow-up (T2) is intended to evaluate the maintenance of treatment effects within the intervention group only; therefore, no between-group comparisons will be conducted at T2. To account for the repeated-measures structure of the data and the correlation among observations across time, analyses will be carried out using mixed-effects models or repeated-measures analysis of variance, depending on the distributional properties of the data. These models will allow the estimation of both within-participant changes over time and between-group differences, while accommodating incomplete data without necessitating case wise deletion. Effect sizes and confidence intervals will be reported to quantify the magnitude and precision of observed differences.

Associations among variables of interest will be explored using multivariate techniques. To better understand the mechanisms underlying treatment effectiveness, mediation analyses will be conducted to examine whether changes in PF or related processes account for improvements in pain acceptance or other clinical outcomes. Moderation analyses will also be performed to identify potential participant-level characteristics that may influence the direction or magnitude of treatment effects.

All analyses will be performed under the intention-to-treat principle whenever feasible, ensuring that participants are analyzed within their originally assigned groups regardless of treatment adherence. Sensitivity analyses will be conducted to evaluate the robustness of the findings under different assumptions regarding missing data handling (e.g., complete-case vs. imputed datasets), model specifications, and adjustment for relevant baseline covariates.

Qualitative data from the clinical interviews will be analyzed using reflexive thematic analysis in accordance with Braun and Clarke's framework (Braun and Clarke, 2006). A primarily inductive analytic approach will be adopted to allow themes to emerge from participants' narratives while remaining sensitive to ACT-related and digital-intervention processes. Data collection will continue until thematic saturation is reached, defined as the point at which no substantially new themes relevant to the research questions are identified in consecutive interviews. Rigor and trustworthiness will be supported through independent coding of a subset of transcripts by at least two researchers, regular analytic discussions, reflexive memoing, documentation of coding decisions in an audit trail and reporting guided by relevant qualitative reporting standards such as COREQ (Tong et al., 2007).

The economic evaluation will be integrated with the clinical data and will rely on the calculation of Quality-Adjusted Life Years (QALYs) derived from EQ-5D-3L utility scores. Incremental cost-effectiveness ratios will be estimated by comparing the costs and outcomes associated with MobACT relative to the waitlist condition, adopting both healthcare-sector and societal perspectives. The uncertainty surrounding these estimates will be examined through non-parametric bootstrapping procedures and appropriate graphical representations, such as cost-effectiveness planes and acceptability curves. The trial protocol and statistical analysis plan will be made publicly accessible through the ClinicalTrial.gov registry (NCT07270588).

## 3 Expected results

Over the past 10 years, ICBT has been developed and tested in several controlled studies, and more recently, multiple studies have implemented Internet-delivered interventions based on ACT principles (Bendelin et al., 2020; Buhrman et al., 2013; Lin et al., 2017; Trompetter et al., 2015). This therapeutic approach, due to its characteristics, may be particularly useful in supporting patients with CP. Hughes, Clark (Hughes et al., 2017) and Veehof, Trompetter (Veehof et al., 2016) emphasize that ACT-based treatments effectively reduce pain intensity, pain interference and emotional distress, while improving patients' QoL.

It is hypothesized that, compared with the waitlist control condition, MobACT will improve pain acceptance at T1 and will produce favorable changes in clinically relevant secondary outcomes, including pain interference, pain intensity, quality of life, sleep quality, psychological flexibility, self-efficacy, coping, anxiety and depressive symptoms. It is also expected that changes in ACT-related processes, particularly psychological flexibility and pain acceptance, will help explain improvements in clinical outcomes, and that the intervention will show a favorable cost-effectiveness profile over the T0–T1 comparison period.

## 4 Strengths and limitations

This study presents several methodological strengths. First, it is a randomized controlled trial evaluating a guided internet-based ACT intervention for adults with chronic pain in Italy, a context in which no internet-delivered ACT programme for CP has yet been formally tested. The intervention is delivered through the secure and evidence-based *Iterapi* platform, which has been widely used for digital mental health interventions and supports standardized treatment delivery. In addition, the study includes a comprehensive assessment of clinical, psychological and economic outcomes, allowing for a multidimensional evaluation of intervention effects, including cost-effectiveness. The use of validated Italian versions of primary and secondary outcome measures further strengthens the methodological rigor and cultural appropriateness of the assessment strategy. However, some limitations should be acknowledged. The waitlist control condition is a relatively weak comparator and does not control for all non-specific factors such as expectancy, therapist contact or digital attention. The delayed-intervention design restricts valid between-group comparisons to the post-intervention assessment (T1), because waitlist participants receive access to MobACT after T1; consequently, no causal between-group inference can be made regarding 6-month maintenance effects. Participants and clinicians cannot be blinded to allocation, and all outcomes are self-reported and administered online, which may introduce expectancy effects, response bias and variability related to pain intensity, emotional state or cognitive load at the time of questionnaire completion. Recruitment conducted entirely online may also limit representativeness by favoring individuals with higher digital literacy, greater motivation for psychological intervention or better access to internet-enabled devices. Finally, because publication of this protocol occurs after trial initiation, the protocol should be interpreted together with the trial registry and ethics documentation; nevertheless, the present manuscript reports no outcome data and clarifies the pre-specified design, outcome hierarchy and analysis plan before trial results are disseminated.

## 5 Data protection and confidentiality

All data collected during the study will be managed in line with the General Data Protection Regulation (GDPR, EU Regulation 2016/679). Data will be stored on encrypted servers through the secure *Iterapi* platform. Personal identifiers will remain strictly separated from all clinical and research data and will be linked only through anonymized participant codes (e.g., 1248cgyy, 1307tzhf). Access to the data will be restricted to authorized members of the research team. Following completion of the trial, data will be retained for a minimum of five years, in accordance with institutional and national guidelines.

## 6 Data monitoring

Throughout the trial, regular internal meetings will be held every three weeks to review recruitment progress, adherence levels, technical aspects of data acquisition and any issues arising during implementation. Deviations from the protocol, if any, will be documented, evaluated and reported according to ethical requirements and best practices for clinical research transparency. Moreover, the *Iterapi* platform automatically records all participant activity, including module access, questionnaire completion and timestamps for each assessment. These built-in monitoring functions allow the research team to identify irregularities, missing data or technical issues in real time. Any inconsistencies or interruptions in the data collection flow will be reviewed by the investigators, who will take appropriate action to ensure continuity and accuracy in data collection.

## 7 Protocol amendments

No substantive changes to the approved study design, intervention, eligibility criteria, outcome hierarchy, assessment schedule or primary endpoint have been introduced as part of the peer-review revision of this protocol manuscript. Any future substantial amendments that may influence participant safety, trial conduct or scientific integrity will be submitted to the Ethics Committee of Universit0 Cattolica del Sacro Cuore, Milan, for approval before implementation. All approved changes and registry clarifications will be documented on ClinicalTrials.gov with the appropriate date and rationale. The present manuscript reflects the protocol version currently approved by the Ethics Committee and registered on ClinicalTrials.gov.

## 8 Interim analysis and stopping guidelines

No interim analyses are planned for this trial, given the low-risk nature of the study. Therefore, no formal stopping guidelines have been established. Nevertheless, the study team will continue to monitor participants' safety throughout the trial, and the intervention delivery may be paused in the case of adverse psychological events.

## 9 Protocol adherence and reporting guidelines

This protocol was developed and reported in accordance with the SPIRIT 2013 guidelines and their 2025 update (Chan et al., 2025). A completed SPIRIT 2025 checklist is provided as supplementary material (see Supplementary file 2).

## 10 Dissemination plan

Study findings will be disseminated via publications in open-access, peer-reviewed journals and presentations at national and international conferences in the fields of health psychology, pain medicine and digital health. Results will be also communicated to patient organizations (particularly the ones involved in study recruitment) and clinical partners using summary reports. To facilitate broader dissemination, lay summaries will be developed for the public and patient communities, including dissemination through social media. Where appropriate, anonymized datasets and statistical analysis code will be made available to researchers upon reasonable request.
