# Supplementary material for: Effectiveness and cost-effectiveness of a guided internet-based Acceptance and Commitment Therapy intervention (MobACT) for adults with Chronic Pain in Italy: study protocol for a randomized controlled trial
Source: Front Psychol. 2026 Jul 13;17:1813251. doi: 10.3389/fpsyg.2026.1813251 (PMC13403208; doi:10.3389/fpsyg.2026.1813251)
Supplement: Supplementary file 1 [file Data_Sheet_1.pdf]

Supplementary Material 1:

Semi-structured post-intervention interview.

Introduction and informed consent

Thank you for participating in this interview. The aim is to understand your experience with the MobACT program. May I record this conversation for research purposes? Your responses will remain confidential. Can I proceed?

Feasibility of the intervention

Access and use of the platform

How did you find accessing the treatment platform? Were there any technical difficulties or barriers that you encountered?

Understanding of the content

How easy was it to understand the materials presented in the modules?

Time management

Did you find it feasible to complete each module within the expected timeframe? How did you organize your time to follow the program?

Satisfaction with the intervention

Evaluation of the content

Which modules did you find most useful or interesting, and why?

What impressed you most about the MobACT intervention? Can you identify one element (a word, an expression, a coping strategy, an exercise, an audio narrative, feedback from your therapist, etc.) and explain why this specific aspect remained memorable to you?

Interaction and support

What was your experience with interacting with the online platform and with your assigned therapist?

Suggestions for improvement

Is there anything you would have liked to change or improve in the modules or in the structure of the program?

Success of the intervention

Impact on chronic pain and social functioning

Have you noticed any changes in your chronic pain since the beginning of the program? In what way has the program influenced the management of your pain and your social functioning?

Application of concepts

On a scale from 1 to 10, how capable do you feel of applying the strategies learned during the program in your daily life? In what way?

Consistency between the presentation of the intervention and the intervention itself

Was the program consistent with what had been presented to you before starting the intervention?

For example, compared with what emerged during the pre-intervention semi-structured interview, or what was shared with you through the chronic pain association, during the presentation by the general practitioner or physiotherapist, through the flyer, or through any other way by which you became aware of the program, did you find correspondence between expectations and reality?

Measurement of success

How would you evaluate the overall success of the program? Did it meet your expectations?

Conclusion

General feedback:

Do you have any additional comments or thoughts you would like to share about the program?

Possible continuation of the intervention

Considering that the aim of the intervention was to improve the management and acceptance of chronic pain, how would you imagine a possible continuation of the pathway in a way that would be useful for you?

Although the intervention ends here for now (except for possible participation in the follow-up phase, if you belong to the experimental group), what would an ideal continuation of this pathway look like for you?

Acknowledgments and next steps

Thank you for taking the time to participate in this interview. Your feedback is very important to us and will be used to improve future programs. Do you have any questions?
